# 4-[4-(Piperidin-1-yl)piperidin-1-yl]benzonitrile

**DOI:** 10.1107/S160053680905507X

**Published:** 2010-01-09

**Authors:** Guo-bin Xu, Jian-you Shi, Li-juan Chen, You-fu Luo

**Affiliations:** a State Key Laboratory of Biotherapy, West China Hospital, West China Medical School, Sichuan University, Chengdu, Sichuan 610041, People’s Republic of China

## Abstract

In the title compound, C_17_H_23_N_3_, both piperidine rings adopt chair conformations. In the crystal packing, intermolecular C—H⋯N hydrogen bonds and C—H⋯π interactions are present.

## Related literature

For general background, see: Pevarello *et al.* (2006 ▶).
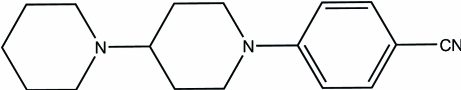


## Experimental

### 

#### Crystal data

C_17_H_23_N_3_
*M* *_r_* = 269.38
Monoclinic, 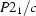

*a* = 10.090 (2) Å
*b* = 11.100 (2) Å
*c* = 13.446 (3) Å
β = 100.72 (3)°
*V* = 1479.7 (5) Å^3^
*Z* = 4
Mo *K*α radiation
μ = 0.07 mm^−1^
*T* = 113 K
0.26 × 0.25 × 0.20 mm

#### Data collection

Rigaku Saturn CCD area-detector diffractometer
Absorption correction: multi-scan (*CrystalClear*; Rigaku/MSC, 2005 ▶) *T*_min_ = 0.981, *T*_max_ = 0.986
11970 measured reflections
3500 independent reflections
2749 reflections with *I* > 2σ(*I*)
*R*_int_ = 0.032

#### Refinement

*R*[*F* ^2^ > 2σ(*F* ^2^)] = 0.038
*wR*(*F* ^2^) = 0.111
*S* = 1.12
3500 reflections
182 parameters
H-atom parameters constrained
Δρ_max_ = 0.27 e Å^−3^
Δρ_min_ = −0.19 e Å^−3^

### 

Data collection: *CrystalClear* (Rigaku/MSC, 2005 ▶); cell refinement: *CrystalClear*; data reduction: *CrystalClear*; program(s) used to solve structure: *SHELXS97* (Sheldrick, 2008 ▶); program(s) used to refine structure: *SHELXL97* (Sheldrick, 2008 ▶); molecular graphics: *ChemBioDraw Ultra* CambridgeSoft (2008 ▶); software used to prepare material for publication: *SHELXL97*.

## Supplementary Material

Crystal structure: contains datablocks global, I. DOI: 10.1107/S160053680905507X/kp2240sup1.cif

Structure factors: contains datablocks I. DOI: 10.1107/S160053680905507X/kp2240Isup2.hkl

Additional supplementary materials:  crystallographic information; 3D view; checkCIF report

## Figures and Tables

**Table 1:** Hydrogen-bond geometry (Å, °) *Cg* is the centroid of the C11–C16 ring.

*D*—H⋯*A*	*D*—H	H⋯*A*	*D*⋯*A*	*D*—H⋯*A*
C6—H6⋯*Cg*^i^	1.00	2.99	3.9363 (14)	158
C16—H16⋯N3^ii^	0.95	2.54	3.3442 (16)	143

Symmetry codes: (i) 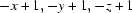; (ii) 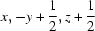.

**Fig. 1.**
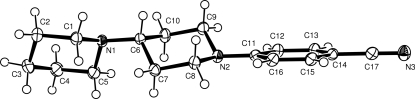
The molecular structure of the title compound, with displacement ellipsoids drawn at the 30% probability level.

**Fig. 2.**
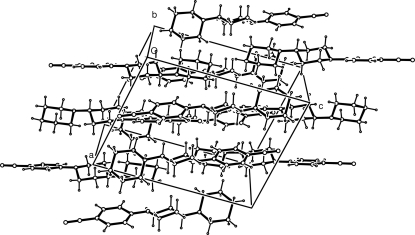
The crystal packing of I dominated by van der Waals interactions.

## supplementary crystallographic information

### Comment

4-(4-(Piperidin-1-yl)piperidin-1-yl)benzonitrile are key intermediates which can be used to synthesize 3-aminopyrazole derivatives, which can be used as precursors for anticancer and  anti-malarial agents. In the structure of the title molecule (Fig. 1) both piperidine rings are in a chair conformation. A crystal packing is dominated by van der Waals interactions (Fig. 2).

### Experimental

A DMSO solution of 1-(piperidin-4-yl)piperidine (4.37 g, 0.01 mol) with 4-fluorobenzonitrile (1.21 g, 0.01 mol) was heated to reflux for 3 h, then water (50 ml) was added into the solution. The mixture was extracted with CH_2_Cl_2_. After  the solvent was removed a red crystalline powder was obtained; its recrystallisation from a methanol solution after 5 days yielded single crystals.

### Crystal data

**Table:**

C_17_H_23_N_3_	*F*(000) = 584
*M**_r_* = 269.38	*D*_x_ = 1.209 Mg m^−^^3^
Monoclinic, *P*2_1_/*c*	Mo *K*α radiation, λ = 0.71073 Å
Hall symbol: -P 2ybc	Cell parameters from 4488 reflections
*a* = 10.090 (2) Å	θ = 2.7–27.9°
*b* = 11.100 (2) Å	µ = 0.07 mm^−^^1^
*c* = 13.446 (3) Å	*T* = 113 K
β = 100.72 (3)°	Block, red
*V* = 1479.7 (5) Å^3^	0.26 × 0.25 × 0.20 mm
*Z* = 4	

### Data collection

**Table:**

Rigaku Saturn CCD area-detector diffractometer	3500 independent reflections
Radiation source: rotating anode	2749 reflections with *I* > 2σ(*I*)
confocal	*R*_int_ = 0.032
Detector resolution: 7.31 pixels mm^-1^	θ_max_ = 27.9°, θ_min_ = 2.8°
ω and φ scans	*h* = −13→10
Absorption correction: multi-scan (*CrystalClear*; Rigaku/MSC, 2005)	*k* = −14→14
*T*_min_ = 0.981, *T*_max_ = 0.986	*l* = −17→17
11970 measured reflections	

### Refinement

**Table:**

Refinement on *F*^2^	Secondary atom site location: difference Fourier map
Least-squares matrix: full	Hydrogen site location: inferred from neighbouring sites
*R*[*F*^2^ > 2σ(*F*^2^)] = 0.038	H-atom parameters constrained
*wR*(*F*^2^) = 0.111	*w* = 1/[σ^2^(*F*_o_^2^) + (0.0612*P*)^2^ + 0.0668*P*] where *P* = (*F*_o_^2^ + 2*F*_c_^2^)/3
*S* = 1.12	(Δ/σ)_max_ < 0.001
3500 reflections	Δρ_max_ = 0.27 e Å^−^^3^
182 parameters	Δρ_min_ = −0.19 e Å^−^^3^
0 restraints	Extinction correction: *SHELXL97* (Sheldrick, 2008), Fc^*^=kFc[1+0.001xFc^2^λ^3^/sin(2θ)]^-1/4^
Primary atom site location: structure-invariant direct methods	Extinction coefficient: 0.033 (7)

### Special details

**Table:**

Geometry. All e.s.d.'s (except the e.s.d. in the dihedral angle between two l.s. planes) are estimated using the full covariance matrix. The cell e.s.d.'s are taken into account individually in the estimation of e.s.d.'s in distances, angles and torsion angles; correlations between e.s.d.'s in cell parameters are only used when they are defined by crystal symmetry. An approximate (isotropic) treatment of cell e.s.d.'s is used for estimating e.s.d.'s involving l.s. planes.
Refinement. Refinement of *F*^2^ against ALL reflections. The weighted *R*-factor *wR* and goodness of fit *S* are based on *F*^2^, conventional *R*-factors *R* are based on *F*, with *F* set to zero for negative *F*^2^. The threshold expression of *F*^2^ > σ(*F*^2^) is used only for calculating *R*-factors(gt) *etc*. and is not relevant to the choice of reflections for refinement. *R*-factors based on *F*^2^ are statistically about twice as large as those based on *F*, and *R*- factors based on ALL data will be even larger.

### Fractional atomic coordinates and isotropic or equivalent isotropic displacement parameters (Å^2^)

**Table:**

	*x*	*y*	*z*	*U*_iso_*/*U*_eq_	
N1	0.19321 (8)	0.59022 (8)	0.73578 (6)	0.0193 (2)	
N2	0.26675 (8)	0.49828 (7)	0.43659 (6)	0.0187 (2)	
N3	0.48688 (9)	0.35177 (8)	0.00345 (7)	0.0256 (2)	
C1	0.21163 (10)	0.49931 (10)	0.81631 (8)	0.0225 (2)	
H1A	0.3069	0.4727	0.8304	0.027*	
H1B	0.1547	0.4283	0.7934	0.027*	
C2	0.17411 (11)	0.54896 (10)	0.91273 (8)	0.0270 (3)	
H2A	0.2363	0.6154	0.9391	0.032*	
H2B	0.1839	0.4848	0.9647	0.032*	
C3	0.02945 (11)	0.59541 (11)	0.89283 (9)	0.0310 (3)	
H3A	−0.0339	0.5270	0.8768	0.037*	
H3B	0.0102	0.6360	0.9542	0.037*	
C4	0.00956 (11)	0.68349 (11)	0.80492 (9)	0.0307 (3)	
H4A	0.0638	0.7569	0.8249	0.037*	
H4B	−0.0865	0.7074	0.7881	0.037*	
C5	0.05156 (10)	0.62790 (11)	0.71205 (8)	0.0274 (3)	
H5A	−0.0062	0.5573	0.6894	0.033*	
H5B	0.0392	0.6875	0.6563	0.033*	
C6	0.24849 (10)	0.54949 (9)	0.64737 (8)	0.0182 (2)	
H6	0.3448	0.5272	0.6733	0.022*	
C7	0.18209 (10)	0.43925 (9)	0.59102 (8)	0.0205 (2)	
H7A	0.1816	0.3721	0.6394	0.025*	
H7B	0.0873	0.4584	0.5608	0.025*	
C8	0.25693 (10)	0.40038 (9)	0.50799 (8)	0.0204 (2)	
H8A	0.3488	0.3734	0.5391	0.024*	
H8B	0.2093	0.3313	0.4708	0.024*	
C9	0.32454 (11)	0.60897 (9)	0.48736 (8)	0.0224 (2)	
H9A	0.3206	0.6742	0.4366	0.027*	
H9B	0.4206	0.5948	0.5172	0.027*	
C10	0.25036 (11)	0.64891 (9)	0.56997 (8)	0.0229 (2)	
H10A	0.1566	0.6711	0.5394	0.027*	
H10B	0.2951	0.7211	0.6042	0.027*	
C11	0.31345 (9)	0.46754 (9)	0.34801 (7)	0.0179 (2)	
C12	0.32252 (10)	0.55520 (9)	0.27405 (8)	0.0209 (2)	
H12	0.2976	0.6360	0.2849	0.025*	
C13	0.36689 (10)	0.52613 (9)	0.18606 (8)	0.0212 (2)	
H13	0.3727	0.5870	0.1374	0.025*	
C14	0.40341 (10)	0.40774 (9)	0.16801 (8)	0.0182 (2)	
C15	0.39452 (10)	0.31974 (9)	0.24037 (8)	0.0203 (2)	
H15	0.4191	0.2390	0.2289	0.024*	
C16	0.35023 (10)	0.34886 (9)	0.32857 (8)	0.0205 (2)	
H16	0.3445	0.2876	0.3770	0.025*	
C17	0.45003 (10)	0.37726 (9)	0.07679 (8)	0.0200 (2)	

### Atomic displacement parameters (Å^2^)

**Table:**

	*U*^11^	*U*^22^	*U*^33^	*U*^12^	*U*^13^	*U*^23^
N1	0.0189 (4)	0.0234 (5)	0.0162 (4)	0.0020 (3)	0.0049 (3)	−0.0006 (3)
N2	0.0253 (4)	0.0146 (4)	0.0169 (4)	−0.0026 (3)	0.0060 (3)	−0.0002 (3)
N3	0.0337 (5)	0.0195 (5)	0.0252 (5)	−0.0013 (4)	0.0101 (4)	−0.0025 (4)
C1	0.0240 (5)	0.0248 (5)	0.0194 (5)	0.0000 (4)	0.0053 (4)	0.0015 (4)
C2	0.0287 (6)	0.0343 (6)	0.0190 (6)	−0.0034 (5)	0.0071 (4)	−0.0014 (5)
C3	0.0272 (6)	0.0414 (7)	0.0273 (6)	−0.0046 (5)	0.0127 (5)	−0.0073 (5)
C4	0.0237 (5)	0.0400 (7)	0.0300 (6)	0.0065 (5)	0.0085 (5)	−0.0051 (5)
C5	0.0215 (5)	0.0370 (6)	0.0237 (6)	0.0071 (5)	0.0044 (4)	−0.0020 (5)
C6	0.0183 (5)	0.0197 (5)	0.0170 (5)	0.0005 (4)	0.0045 (4)	−0.0008 (4)
C7	0.0226 (5)	0.0200 (5)	0.0196 (5)	−0.0024 (4)	0.0058 (4)	0.0011 (4)
C8	0.0272 (5)	0.0160 (5)	0.0189 (5)	−0.0022 (4)	0.0069 (4)	0.0008 (4)
C9	0.0287 (5)	0.0187 (5)	0.0210 (5)	−0.0062 (4)	0.0076 (4)	−0.0031 (4)
C10	0.0315 (6)	0.0177 (5)	0.0212 (6)	−0.0024 (4)	0.0094 (4)	−0.0023 (4)
C11	0.0178 (5)	0.0183 (5)	0.0170 (5)	−0.0011 (4)	0.0018 (4)	−0.0009 (4)
C12	0.0263 (5)	0.0161 (5)	0.0209 (5)	0.0028 (4)	0.0059 (4)	0.0000 (4)
C13	0.0268 (5)	0.0187 (5)	0.0185 (5)	0.0012 (4)	0.0054 (4)	0.0027 (4)
C14	0.0196 (5)	0.0181 (5)	0.0171 (5)	−0.0009 (4)	0.0033 (4)	−0.0014 (4)
C15	0.0238 (5)	0.0164 (5)	0.0205 (6)	0.0007 (4)	0.0033 (4)	−0.0017 (4)
C16	0.0253 (5)	0.0170 (5)	0.0187 (5)	−0.0007 (4)	0.0031 (4)	0.0011 (4)
C17	0.0236 (5)	0.0145 (5)	0.0216 (6)	−0.0012 (4)	0.0038 (4)	0.0000 (4)

### Geometric parameters (Å, °)

**Table:**

N1—C5	1.4663 (13)	C6—H6	1.0000
N1—C1	1.4664 (13)	C7—C8	1.5219 (14)
N1—C6	1.4749 (13)	C7—H7A	0.9900
N2—C11	1.4020 (13)	C7—H7B	0.9900
N2—C8	1.4656 (13)	C8—H8A	0.9900
N2—C9	1.4723 (13)	C8—H8B	0.9900
N3—C17	1.1520 (13)	C9—C10	1.5164 (14)
C1—C2	1.5200 (14)	C9—H9A	0.9900
C1—H1A	0.9900	C9—H9B	0.9900
C1—H1B	0.9900	C10—H10A	0.9900
C2—C3	1.5241 (16)	C10—H10B	0.9900
C2—H2A	0.9900	C11—C16	1.4063 (14)
C2—H2B	0.9900	C11—C12	1.4064 (14)
C3—C4	1.5183 (17)	C12—C13	1.3788 (14)
C3—H3A	0.9900	C12—H12	0.9500
C3—H3B	0.9900	C13—C14	1.3981 (14)
C4—C5	1.5220 (15)	C13—H13	0.9500
C4—H4A	0.9900	C14—C15	1.3933 (14)
C4—H4B	0.9900	C14—C17	1.4335 (14)
C5—H5A	0.9900	C15—C16	1.3811 (14)
C5—H5B	0.9900	C15—H15	0.9500
C6—C10	1.5195 (14)	C16—H16	0.9500
C6—C7	1.5269 (14)		
			
C5—N1—C1	109.98 (8)	C8—C7—H7A	109.4
C5—N1—C6	114.29 (8)	C6—C7—H7A	109.4
C1—N1—C6	111.67 (8)	C8—C7—H7B	109.4
C11—N2—C8	116.77 (8)	C6—C7—H7B	109.4
C11—N2—C9	115.49 (8)	H7A—C7—H7B	108.0
C8—N2—C9	112.59 (8)	N2—C8—C7	111.93 (8)
N1—C1—C2	111.27 (9)	N2—C8—H8A	109.2
N1—C1—H1A	109.4	C7—C8—H8A	109.2
C2—C1—H1A	109.4	N2—C8—H8B	109.2
N1—C1—H1B	109.4	C7—C8—H8B	109.2
C2—C1—H1B	109.4	H8A—C8—H8B	107.9
H1A—C1—H1B	108.0	N2—C9—C10	112.14 (8)
C1—C2—C3	110.81 (9)	N2—C9—H9A	109.2
C1—C2—H2A	109.5	C10—C9—H9A	109.2
C3—C2—H2A	109.5	N2—C9—H9B	109.2
C1—C2—H2B	109.5	C10—C9—H9B	109.2
C3—C2—H2B	109.5	H9A—C9—H9B	107.9
H2A—C2—H2B	108.1	C9—C10—C6	111.10 (8)
C4—C3—C2	109.76 (9)	C9—C10—H10A	109.4
C4—C3—H3A	109.7	C6—C10—H10A	109.4
C2—C3—H3A	109.7	C9—C10—H10B	109.4
C4—C3—H3B	109.7	C6—C10—H10B	109.4
C2—C3—H3B	109.7	H10A—C10—H10B	108.0
H3A—C3—H3B	108.2	N2—C11—C16	121.86 (9)
C3—C4—C5	111.16 (10)	N2—C11—C12	120.57 (9)
C3—C4—H4A	109.4	C16—C11—C12	117.55 (9)
C5—C4—H4A	109.4	C13—C12—C11	121.27 (9)
C3—C4—H4B	109.4	C13—C12—H12	119.4
C5—C4—H4B	109.4	C11—C12—H12	119.4
H4A—C4—H4B	108.0	C12—C13—C14	120.46 (9)
N1—C5—C4	110.24 (9)	C12—C13—H13	119.8
N1—C5—H5A	109.6	C14—C13—H13	119.8
C4—C5—H5A	109.6	C15—C14—C13	118.98 (9)
N1—C5—H5B	109.6	C15—C14—C17	120.39 (9)
C4—C5—H5B	109.6	C13—C14—C17	120.63 (9)
H5A—C5—H5B	108.1	C16—C15—C14	120.57 (9)
N1—C6—C10	112.59 (8)	C16—C15—H15	119.7
N1—C6—C7	116.67 (8)	C14—C15—H15	119.7
C10—C6—C7	107.60 (8)	C15—C16—C11	121.17 (9)
N1—C6—H6	106.4	C15—C16—H16	119.4
C10—C6—H6	106.4	C11—C16—H16	119.4
C7—C6—H6	106.4	N3—C17—C14	179.37 (11)
C8—C7—C6	111.06 (8)		
			
C5—N1—C1—C2	−60.67 (11)	N2—C9—C10—C6	56.38 (12)
C6—N1—C1—C2	171.35 (8)	N1—C6—C10—C9	172.41 (8)
N1—C1—C2—C3	56.80 (12)	C7—C6—C10—C9	−57.65 (11)
C1—C2—C3—C4	−52.73 (12)	C8—N2—C11—C16	−0.48 (13)
C2—C3—C4—C5	53.76 (12)	C9—N2—C11—C16	135.34 (10)
C1—N1—C5—C4	60.92 (12)	C8—N2—C11—C12	178.25 (9)
C6—N1—C5—C4	−172.56 (9)	C9—N2—C11—C12	−45.93 (12)
C3—C4—C5—N1	−58.25 (12)	N2—C11—C12—C13	−179.30 (9)
C5—N1—C6—C10	63.26 (11)	C16—C11—C12—C13	−0.52 (14)
C1—N1—C6—C10	−171.10 (8)	C11—C12—C13—C14	0.37 (15)
C5—N1—C6—C7	−61.87 (12)	C12—C13—C14—C15	−0.13 (15)
C1—N1—C6—C7	63.77 (11)	C12—C13—C14—C17	−179.78 (9)
N1—C6—C7—C8	−174.76 (8)	C13—C14—C15—C16	0.05 (15)
C10—C6—C7—C8	57.63 (11)	C17—C14—C15—C16	179.70 (9)
C11—N2—C8—C7	−169.71 (8)	C14—C15—C16—C11	−0.21 (15)
C9—N2—C8—C7	53.24 (11)	N2—C11—C16—C15	179.21 (9)
C6—C7—C8—N2	−56.30 (11)	C12—C11—C16—C15	0.44 (15)
C11—N2—C9—C10	169.02 (8)	C15—C14—C17—N3	38 (9)
C8—N2—C9—C10	−53.35 (11)	C13—C14—C17—N3	−142 (9)

### Hydrogen-bond geometry (Å, °)

**Table:**

Cg is the centroid of the C11–C16 ring.

**Table:**

*D*—H···*A*	*D*—H	H···*A*	*D*···*A*	*D*—H···*A*
C6—H6···Cg^i^	1.00	2.99	3.9363 (14)	158
C16—H16···N3^ii^	0.95	2.54	3.3442 (16)	143

Symmetry codes: (i) −*x*+1, −*y*+1, −*z*+1; (ii) *x*, −*y*+1/2, *z*+1/2.
